# Supplementary material for: LTA4H extensively associates with mRNAs and lncRNAs indicative of its novel regulatory targets
Source: PeerJ. 2023 Mar 10;11:e14875. doi: 10.7717/peerj.14875 (PMC10010175; doi:10.7717/peerj.14875)
Supplement: File S2 [file peerj-11-14875-s005.zip › Raw data files/Motif_results/piranha/LTA4H_IP_1_vs_LTA4H_input_1/homerResults.html]

.// - Homer de novo Motif Results


# Homer *de novo* Motif Results (.//)

Known Motif Enrichment Results  
Gene Ontology Enrichment Results  
If Homer is having trouble matching a motif to a known motif, try copy/pasting the matrix file into
STAMP  
More information on motif finding results: HOMER
| Description of Results
| Tips
  
Total target sequences = 8558  
Total background sequences = 71970  
\* - possible false positive  

|  |  |  |  |  |  |  |  |  |
| --- | --- | --- | --- | --- | --- | --- | --- | --- |
| Rank | Motif | P-value | log P-pvalue | % of Targets | % of Background | STD(Bg STD) | Best Match/Details | Motif File |
| 1 |  | 1e-186 | -4.302e+02 | 20.33% | 9.16% | 17.7bp (23.3bp) | hsa-miR-548ag MIMAT0018969 Homo sapiens miR-548ag Targets (miRBase)(0.712) More Information \| Similar Motifs Found | motif file (matrix) |
| 2 |  | 1e-61 | -1.425e+02 | 20.07% | 13.16% | 19.1bp (28.2bp) | hsa-miR-4778-3p MIMAT0019937 Homo sapiens miR-4778-3p Targets (miRBase)(0.729) More Information \| Similar Motifs Found | motif file (matrix) |
| 3 |  | 1e-57 | -1.334e+02 | 9.40% | 4.89% | 15.8bp (24.5bp) | hsa-miR-425\* MIMAT0001343 Homo sapiens miR-425\* Targets (miRBase)(0.714) More Information \| Similar Motifs Found | motif file (matrix) |
| 4 |  | 1e-54 | -1.265e+02 | 9.96% | 5.40% | 16.0bp (21.1bp) | hsa-miR-4766-5p MIMAT0019917 Homo sapiens miR-4766-5p Targets (miRBase)(0.681) More Information \| Similar Motifs Found | motif file (matrix) |
| 5 |  | 1e-50 | -1.159e+02 | 3.41% | 1.11% | 28.9bp (31.2bp) | hsa-miR-297 MIMAT0004450 Homo sapiens miR-297 Targets (miRBase)(0.727) More Information \| Similar Motifs Found | motif file (matrix) |
| 6 |  | 1e-31 | -7.188e+01 | 16.87% | 12.24% | 19.6bp (26.5bp) | hsa-miR-572 MIMAT0003237 Homo sapiens miR-572 Targets (miRBase)(0.713) More Information \| Similar Motifs Found | motif file (matrix) |
| 7 |  | 1e-23 | -5.404e+01 | 3.35% | 1.65% | 15.2bp (27.8bp) | hsa-miR-450a MIMAT0001545 Homo sapiens miR-450a Targets (miRBase)(0.594) More Information \| Similar Motifs Found | motif file (matrix) |
| 8 |  | 1e-20 | -4.656e+01 | 3.86% | 2.12% | 16.9bp (22.8bp) | hsa-miR-361-3p MIMAT0004682 Homo sapiens miR-361-3p Targets (miRBase)(0.743) More Information \| Similar Motifs Found | motif file (matrix) |
| 9 |  | 1e-20 | -4.645e+01 | 12.22% | 9.00% | 15.4bp (21.7bp) | hsa-miR-186\* MIMAT0004612 Homo sapiens miR-186\* Targets (miRBase)(0.524) More Information \| Similar Motifs Found | motif file (matrix) |
| 10 |  | 1e-19 | -4.409e+01 | 18.56% | 14.75% | 16.7bp (18.8bp) | hsa-miR-545 MIMAT0003165 Homo sapiens miR-545 Targets (miRBase)(0.774) More Information \| Similar Motifs Found | motif file (matrix) |
| 11 |  | 1e-17 | -4.103e+01 | 11.64% | 8.69% | 22.0bp (23.2bp) | hsa-miR-625 MIMAT0003294 Homo sapiens miR-625 Targets (miRBase)(0.718) More Information \| Similar Motifs Found | motif file (matrix) |
| 12 |  | 1e-15 | -3.579e+01 | 7.01% | 4.88% | 19.9bp (28.9bp) | hsa-miR-3615 MIMAT0017994 Homo sapiens miR-3615 Targets (miRBase)(0.729) More Information \| Similar Motifs Found | motif file (matrix) |
| 13 |  | 1e-15 | -3.545e+01 | 5.25% | 3.43% | 22.1bp (21.5bp) | hsa-miR-1193 MIMAT0015049 Homo sapiens miR-1193 Targets (miRBase)(0.751) More Information \| Similar Motifs Found | motif file (matrix) |
| 14 |  | 1e-14 | -3.313e+01 | 3.26% | 1.91% | 19.1bp (25.8bp) | hsa-miR-195 MIMAT0000461 Homo sapiens miR-195 Targets (miRBase)(0.739) More Information \| Similar Motifs Found | motif file (matrix) |
| 15 |  | 1e-14 | -3.309e+01 | 16.49% | 13.37% | 15.4bp (19.5bp) | hsa-miR-4261 MIMAT0016890 Homo sapiens miR-4261 Targets (miRBase)(0.758) More Information \| Similar Motifs Found | motif file (matrix) |
| 16 |  | 1e-14 | -3.226e+01 | 1.85% | 0.89% | 23.9bp (23.1bp) | hsa-miR-4782-5p MIMAT0019944 Homo sapiens miR-4782-5p Targets (miRBase)(0.771) More Information \| Similar Motifs Found | motif file (matrix) |
| 17 \* |  | 1e-11 | -2.710e+01 | 0.35% | 0.05% | 12.9bp (34.0bp) | hsa-miR-3913-3p MIMAT0019225 Homo sapiens miR-3913-3p Targets (miRBase)(0.641) More Information \| Similar Motifs Found | motif file (matrix) |
| 18 \* |  | 1e-9 | -2.300e+01 | 4.35% | 3.01% | 16.7bp (18.6bp) | hsa-miR-4444 MIMAT0018962 Homo sapiens miR-4444 Targets (miRBase)(0.630) More Information \| Similar Motifs Found | motif file (matrix) |
| 19 \* |  | 1e-8 | -2.064e+01 | 6.11% | 4.60% | 17.1bp (21.2bp) | hsa-miR-1258 MIMAT0005909 Homo sapiens miR-1258 Targets (miRBase)(0.726) More Information \| Similar Motifs Found | motif file (matrix) |
| 20 \* |  | 1e-7 | -1.823e+01 | 0.18% | 0.02% | 10.1bp (6.5bp) | hsa-miR-626 MIMAT0003295 Homo sapiens miR-626 Targets (miRBase)(0.737) More Information \| Similar Motifs Found | motif file (matrix) |
| 21 \* |  | 1e-6 | -1.530e+01 | 0.44% | 0.16% | 16.7bp (16.7bp) | hsa-miR-4785 MIMAT0019949 Homo sapiens miR-4785 Targets (miRBase)(0.706) More Information \| Similar Motifs Found | motif file (matrix) |
